# Characterization of *Bacillus pumilus* Strains with Targeted Gene Editing for Antimicrobial Peptides and Sporulation Factor

**DOI:** 10.3390/microorganisms11061508

**Published:** 2023-06-06

**Authors:** Iuliia V. Danilova, Iuliia A. Vasileva, Ajgul I. Gilmutdinova, Ilona V. Dyadkina, Liya K. Khusnullina, Damir I. Khasanov, Natalia L. Rudakova, Margarita R. Sharipova

**Affiliations:** Research Laboratory “Agrobioengineering”, Institute of Fundamental Medicine and Biology, Kazan (Volga Region) Federal University, 420008 Kazan, Russia; vasileva891@mail.ru (I.A.V.); aigwinrygilmyizn@gmail.com (A.I.G.); iolantalisof@gmail.com (I.V.D.); liya.xusnullina@bk.ru (L.K.K.); hasda2149@gmail.com (D.I.K.); natalialrudakova@mail.ru (N.L.R.); marsharipova@gmail.com (M.R.S.)

**Keywords:** *Bacillus pumilus*, CRISPR-CAS9, antimicrobial peptides, sporulation factor, antagonistic activity

## Abstract

Due to their capacity to produce antimicrobial peptides that can prevent the growth of diseases, many *Bacillus* spp. are beneficial to plants. In this study, we looked into the antagonistic activity of the *B. pumilus* 3-19 strain and its derivatives following targeted genome editing. Two peptide genes with antibacterial action, bacilysin (*bac*) and bacteriocin (*bact*), and the *sig*F gene, which encodes the sigma factor of sporulation, were specifically inactivated using the CRISPR-Cas9 system in the genome of *B. pumilus* 3-19. Antibacterial activity against *B. cereus* and *Pantoea brenneri* decreased as a result of the inactivation of target genes in the *B. pumilus* 3-19 genome, with a noticeable effect against bacilysin. The growth dynamics of the culture changed when the *bac*, *bact*, and *sig*F genes were inactivated, and the altered strains had less proteolytic activity. An asporogenic mutant of *B. pumilus* 3-19 was obtained by inactivating the *sig*F gene. It has been proven that bacilysin plays a unique part in the development of *B. pumilus* 3-19’s antagonistic action against soil microorganisms.

## 1. Introduction

Research on the mechanisms of interaction between microorganisms and plants is advancing as interest in the use of microbes with advantageous qualities in agriculture grows. The positive impact of plant rhizosphere bacteria on plant growth and yield has been shown by numerous researchers [1,2,3,4,5]. These bacteria are referred to as plant growth promotion rhizobacteria (PGPR) [6]. Bacterial strains from the genera *Bacillus*, *Pseudomonas*, and *Agrobacterium* contribute most to biological control as soil- and plant-associated microbes. The main type of rhizobacteria that form spores that can survive in soil for a long period of time under harsh environmental conditions and are able to secrete metabolites that stimulate plant growth and prevent pathogen infestation belong to the *Bacillus* species [4]. The most important feature of the members of the *Bacillus* species is their diverse secondary metabolism and ability to produce a wide variety of structurally different antagonistic substances. In the *B. subtilis* genome, from 4 to 5% of the genes are intended for the synthesis of secondary metabolites capable of producing more than 24 structurally diverse antimicrobial compounds [7]. Another typical isolate is the plant-associated strain *B. amyloliquefaciens* FZB42, which produces secondary metabolites such as bacteriocins, antimicrobial peptides and lipopeptides, polyketides, and siderophores, while also possessing growth-promoting properties for plants [8].

Antimicrobial peptides (AMPs) of *Bacillus* sp. can be divided into two subgroups, depending on the synthesis pathway. The first group includes small microbial peptides that are synthesized by large enzymatic complexes [9]. For example, the species *Brevibacillus brevis* produces small cyclic peptides thyrocidin and gramicidin S. At the same time, *B. licheniformis* synthesizes bacitracin A, which consists of 12 amino acids [9]. Another characteristic example is bacilysin, which is a small peptide containing an N-terminal alanine and L-anticapsin residue; for example, it can be produced by *B. subtilis*, *B. pumilus*, and *B. amyloliquefaciens* [10]. The biosynthesis of this AMP occurs with the participation of an amino acid ligase (bacilysin synthetase) in the presence of ATP and Mg^2+^ [9]. It has recently been shown that the bacilysin-deleted strain is capable of expressing a wide range of proteins. It has been demonstrated that bacilysin act directly or indirectly as a small pleiotropic signaling molecule affecting various cellular functions including spore quality [11].

The second subgroup of antimicrobial peptides includes ribosomal-synthesized peptides named bacteriocins. They contain 12 to 50 amino acid residues and are usually cationic. Bacteria produce bacteriocins against strains closely related to the producer. This group of peptide antibiotics is heterogeneous, amphiphilic, and/or hydrophobic [9].

An in silico analysis of the genome of *B. pumilus* 3-19 strains showed that it contains the genes responsible for the synthesis of these antimicrobial peptides [12]. In this study, the inactivation of the genes of antimicrobial peptides—bacilysin and bacteriocin—in the *B. pumilus* 3-19 genome using the CRISPR-Cas genome editing system will be carried out in order to determine the contribution of each of them to the antagonistic activity of the strain.

Studies on biological control of bacterial plant pathogens are generally sparse and cover only a few bacterial species. Many authors have pointed to the benefits of surfactin as a surfactant [13]. However, several studies have shown a protective role for this lipopeptide compound. Bayes et al. suggested that surfactin has a protective effect on *Arabidopsis* roots against the phytopathogenic bacterium *Pseudomonas syringae* by destroying its cell membrane, while Hinarejos et al. described the efficacy of *B. subtilis* IAB/BS03 surfactin and iturin A against *P. syringae* [14,15]. In addition, a lipopeptide extract containing iturin and surfactin isolated from *B. subtilis* showed cell wall degradation in *Xanthomonas campestris pv. campestris* and *X. axonopodis pv. citri* [16]. In addition, several studies have observed similar results for *B. amyloliquefaciens* lipopeptides against bacterial plant pathogens.

It has recently been found that in *B. subtilis*, many physiological processes, such as sporulation, biofilm formation, competence development, proteolytic enzyme synthesis, and production of antimicrobial peptides, are controlled by a quorum-sensing mechanism (by ComQXPA). The critical level of accumulated extracellular peptide pheromones (ComX), competence factors, and sporulation causes a regulatory response of genes that depends on population density. Studies show that the activation of the *bac*ABCDE operon is tightly regulated by four proteins: AbrB and CodY carry out negative regulation of transcription, while ComA and Spo0A are positive (the main regulator of sporulation entry). Thus, the deletion of the *spo*0A gene led to a sharp decrease in the synthesis of bacilysin. It has recently been shown that the bacilysin-deleted strain is capable of expressing a wide range of proteins [11]. Therefore, another goal of this study was to inactivate the sporulation factor (*sig*F) gene in the *B. pumilus* 3-19 genome using the CRISPR-Cas genome editing system and further study the antagonistic activity of the resulting mutant.

Thus, to determine the role of AMPs in the antagonistic activity of *B. pumilus* 3-19, in this work, deletion mutants for the genes of bacilysin (*bac*), bacteriocin (*bact*), and sigma factor sporulation (*sig*F) will be obtained.

## 2. Materials and Methods

### 2.1. Bacterial Strains and Plasmids

The strains used in the work are presented in Table 1.

Plasmid pJOE9282.1 was used in the work (Dr. Prof. J. Altenbuchner, Institute of Industrial Genetics, University of Stuttgart, Stuttgart, Germany) [19].

### 2.2. Media and Growth Conditions

LB agar, LB broth, and Spizizen minimal nutrient medium (if necessary, with the addition of 2% agar) were used as nutrient media [20]. Deep cultivation of cultures was carried out in a thermostat (Inkubations-Schüttelschrank BS4, Braun, Germany) in LB medium (1:7) with a swing of 180 rpm at 37 °C.

Transformation of recombinant DNA into *E. coli* DH5α cells was performed using CaCl2 [21]. *B. pumilus* 3-19 cells were transformed using AEB1 electroporation buffer pH 6.0 [22].

The antibiotics streptomycin and kanamycin were used at concentrations of 500 µg/mL and 15 µg/mL, respectively (PanEco, Moscow, Russia).

The dynamics of the growth of wild and mutant strains was carried out for 83 h in 500 mL flasks at a temperature of +37 °C with a swing of 180 rpm.

Targeted gene inactivation was carried out at 30 °C for 2–3 days on LA [20] medium supplemented with xylose (0.2%) (Chimmed Group, Moscow, Russia) and the antibiotic kanamycin. To inactivate the CRISPR/Cas9 plasmid, selected mutant colonies were cultured in LA medium at 42 °C for 1–2 days. The antimicrobial activity of the original and mutant strains was carried out on LA medium at a temperature of 37 °C.

### 2.3. Plasmid Construction

CRISPR-Cas9 (clustered regularly interspaced short palindromic repeats, CRISPR-associated protein 9) genome editing technology is a modern method for making changes to the genome of bacteria, plants, various cell lines, individual cells, and tissues. Since 2012, the basic CRISPR-Cas9 technology based on Streptococcus pyogenes bacteria has been developed and widely used [23]. The system consists of a Cas9 protein with nuclease activity and two non-coding RNAs (CRISPR-RNA/crRNA and transactivating CRISPR-RNA/tracrRNA) that provide targeted activity of the Cas9 protein towards a specific DNA region. For convenience, both RNA molecules are combined into a common molecule (synthetic single-guide RNA/sgRNA). Thus, the Cas9-sgRNA complex containing 17–30 bp in each homologous region is able to recognize a specific region in the genome and introduce a double-strand break in DNA. Another important component of the CRISPR-Cas9 editing system is the presence of a short conserved sequence named PAM (protospacer adjacent motif) which is located next to the target genome fragment. For the Cas9 nuclease of S. pyogenes PAM, the sequence is NNG. The advantage of using CRISPR-Cas9 technology is the possibility of using several sgRNA molecules that direct the Cas9 nuclease to several regions of the genome at once. Double-strand breaks in DNA formed by CRISPR-Cas9 can be repaired using two mechanisms—non-homologous end joining (NHEJ) and homologous reparations (homologous recombination, HR). Non-homologous repair often results in the formation of deletions or insertions (insertion or deletion/indels) in the target DNA region. Homologous recombination, on the contrary, leads to the exact restoration of DNA relative to the homologous sequence [24].

Joseph Altenbuchner presented a single plasmid system that allows efficient editing of the *B. subtilis* genome [25]. The shuttle vector pJOE9282.1 containing the CRISPR/Cas9 system for target gene inactivation was used in the work. The plasmid (pJOE9282.1) contains the replicon pUC (for *E. coli*) and pE194ts (temperature-sensitive origin for *B. pumilus*), as well as the kanamycin resistance gene (*kan*R). The *cas*9 gene is under the control of the xylose-inducible promoter (P*xyl*), and sgRNA synthesis is regulated by the P*vanP* promoter [19].

In this work, we used the shuttle vector pJOE9282.1, cloned in *E. coli* DH5α cells and containing the CRISPR/Cas9 system, for further inactivation of target genes [19]. Plasmid isolation was performed using a commercial GeneJET Plasmid Miniprep Kit (ThermoFisher Scientific, Waltham, MA, USA).

Gene numbers and their sequences were taken from the genome deposited in the database under the numbers: bacilysin (*bac*-/locus_tag = “GZ55_04230”), bacteriocin (*bact*-/locus_tag = “GZ55_09505”), and sigma factor sporulation (*sigF*-/locus_tag = “GZ55_10725”).

Restriction and ligation of DNA was carried out in accordance with the recommendations of enzyme manufacturers using BsaI restrictase (New England Biolabs, Ipswich, MA, USA), Antarctic Phosphatase (New England BioLabs, Ipswich, MA, USA), and T4 DNA ligase (Thermo Fisher Scientific, Waltham, MA, USA).

The gene sequence for searching of the PAM motif was loaded into the Cas-Designer program (http://www.rgenome.net/, accessed on 25 April 2021). To write primers to sgRNA, 20 bp was extraced before or after the selected PAM motif. The BsaI restriction site was additionally added to the sgRNA primers. Primers for R and L fragments of genes were made in Primer-Blast program (https://www.ncbi.nlm.nih.gov/tools/primer-blast/, accessed on 28 April 2021).

Primer hybridization (Table 2) was carried out in a volume of 10 μL according to the program: 5 min—100 °C; cooling at room temperature overnight. The concentration of primers for hybridization was 100 pmol/µL.

PCR to obtain flanking target gene sequences from *B. pumilus* 3-19 genomic DNA was performed using Phusion polymerase (Phusion High-Fidelity DNA Polymerase, New England Biolabs, Ipswich, MA, USA). The sequences of primer pairs used are presented in Table 3. Primer synthesis was carried out by Evrogen (Moscow, Russia). DNA electrophoresis was performed in 1% agarose gel in Tris-acetate buffer (PanEco, Moscow, Russia). The 1 Kb kit from Fermentas was used as markers (Fermentas, Vilnius, Lithuania).

The resulting gene fragments were inserted into the pJOE9282.1-sgRNA vector at the SfiI site. Construct integrity was verified by sequencing using T7promoter-For and sgRNA-Rev primers for each construct, respectively. Clone Manager 9 software was used to process the sequencing results.

The primer pair cas9-For and cas9-Rev was used to test *B. pumilus* 3-19 for the presence of the resulting constructs containing the CRISPR/Cas9 system (Table 3).

### 2.4. Dynamics of Growth and Sporulation, Proteolytic and Antagonistic Activities of the Studied Strains

The growth dynamics of wild and mutant strains was carried out for 83 h. The strains were cultivated in a thermostat at a temperature of 37 °C with a swing of 180 rpm. An amount of 500 µL of 12 h inoculum was used as inoculum, which was added to 150 mL of LB nutrient medium. Cultivation was carried out in a 500 mL flask.

Samples of 500 μL were used every 2 h for further measurement of proteolytic activity and sporulation. Analysis of the growth of the studied bacteria was performed by changing the optical density of the culture on an xMark spectrophotometer (BioRad) at a wavelength of λ = 590 nm. The amount of biomass was expressed in units of optical density.

Proteolytic activity was determined by the breakdown of azocasein (Sigma, St. Louis, MI, USA) at a concentration of 10 mg/mL dissolved in 0.05 M Tris-HCl buffer, pH 7.3 (PanEco, Moscow, Russia) [26].

The number of spores was counted using the Dorner method (A1) for staining endospores [27]. The number of free spores was expressed as a percentage of the total number of vegetative and sporulating cells (100%), which were counted in phase-contrast microscopy mode (Carl Zeiss Jena microscope) at a magnification of 1600 times in 5 fields of view.

Antimicrobial activity was determined by the method of perpendicular strokes [28]. The test strain was streaked vertically on a Petri dish with LA nutrient medium and grown for a day at 37 °C. Next, test strains were added to the obtained microorganism with a horizontal stroke and incubated for 1 day under conditions optimal for growth. For control, horizontally seeded test strains without the test microorganism were used. After the expiration of the incubation period, antimicrobial activity in the test strain was judged by the size of the zones of inhibition of the test strains. The zone of inhibition of the test strains was measured using a ruler and expressed in mm.

### 2.5. Mathematical Processing of Results

All analyses were performed on three biological replicates. The obtained data were processed using Statgraphics Plus 5.0. and GraphPad Prism 8 statistical software and are presented as the mean ± standard deviation (SD). Student’s *t*-test analysis, analysis of variance (ANOVA) [29], and Tukey’s test were used to calculate the data variance, with *p* < 0.05 representing a significant difference. 

## 3. Results and Discussion

### 3.1. Obtaining Plasmids Carrying Fragments of the Bacilysin, Bacteriocin, and Sporulation Factor Sigma-F Genes Based on the Shuttle Vector pJOE9282.1

Plasmid pJOE9282.1 (~9059 bp) was isolated from *E. coli* DH5α cells and linearized with BsaI.

Next, plasmids carrying fragments of the genes of bacilysin, bacteriocin, and sporulation factor sigma-F were constructed. At the first stage, pJOE9282.1 vector and prehybridized spacer fragments (sgRNA) were ligated.

At the second stage, gene fragments of bacilysin, bacteriocin, and sigma-F sporulation factor amplified with *B.pumilus* 3-19 genomic DNA were inserted into the cloned pJOE9282.1-sgRNA vectors at the SfiI site (Figure S1). The sizes of the PCR products corresponded to the length of the gene fragments: *bac* (L~404 bp and R~402 bp), *bact* (L~501 bp and R~503 bp), and *sigF* (L~500 bp and R~716 bp) (Figure S2).

The resulting plasmids, pDIb11.21, pVYb11.21, and pGAs11.21, with fragments of the bacilysin (*bac*), bacteriocin (*bact*), and sporulation factor (*sigF*) genes, respectively, were cloned in *E. coli* DH5α cells. Transformed *E. coli* DH5α cells were measured for the presence of plasmids pDIb11.21, pVYb11.21, and pGAs11.21 using PCR analysis.

These plasmids carry the gene for the Cas protein, which targets the protospacer (target gene) via targeting gRNA and creates a double-strand break. Further, chromosomal DNA undergoes the HDR (homology-directed repair) repair mechanism, or homologous recombination. This mechanism requires the presence of a DNA template [25]. In the resulting plasmids, the –R and –L gene fragments act as homologous DNA templates. The integrity of the resulting vectors was confirmed by sequencing.

As a result, we obtained plasmids containing the CRISPR/Cas9 system, target DNA sequence (protospacer), and a DNA template for homologous repair—fragments of the bacilysin gene *bac*-L and *bac*-R (pDIb11.21), fragments of the bacteriocin gene *bact*-L and *bact*-R (pVYb11.21), and fragments of the sporulation factor gene *sigF*-L and *sigF*-R (pGAs11.21).

### 3.2. Transformation of B. pumilus Cells with Obtained Plasmids

The resulting plasmids (pDIb11.21, pVYb11.21, and pGAs11.21) were transformed into *B. pumilus* 3-19 cells using an optimized electroporation method [22]. Transformant colonies are shown in Figure S3.

The transformation efficiency in the case of the pDIb11.21 vector was ~121 transformants/μg DNA, that of the pVYb11.21 vector was ~155 transformants/μg DNA, and that of the pGAs11.21 vector was ~119.45 transformants/μg DNA.

### 3.3. Targeted Inactivation of the bac, bact, and sigF Genes by CRISPR/Cas9 Editing

**Figure 1:**
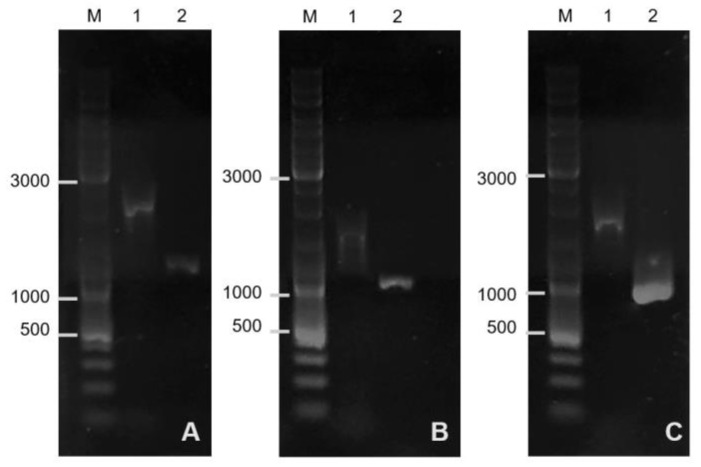
Electrophoresis of amplification products of *bac*, *bact*, and *sigF* gene fragments (PCR from colonies of *B. pumilus* 3-19 and its mutants). (**A**): 1—*B. pumilus* 3-19 (1751 bp), 2—Δ*sigF* (1216 bp); (**B**): 1—*B. pumilus* 3-19 (1751 bp), 2—Δ*bact* (1004 bp); (**C**): 1—*B. pumilus* 3-19 (1751 bp), 2—Δ*bac* (806 bp). M, DNA length marker GeneRuler DNA Ladder Mix.

The selected colonies of transformants were further used for inactivation of the *bac*, *bact*, and *sigF* genes, which was carried out at 30 °C on LA medium with the addition of streptomycin and 0.2% xylose. Colonies appearing within 2 days were placed on LA medium without antibiotic and incubated at 42 °C. Increasing the temperature leads to the loss of the plasmid. This is due to the fact that this plasmid has a temperature-sensitive origin of replication pE194ts. The resulting colonies were selected for subsequent isolation of genomic DNA required for PCR using the following pairs of primers: *bac*-L-For-*bac*-R-Rev; *bact*-L-For-*bact*-R-Rev; *sigF*-L-For-*sigF*-R-Rev. The results are shown in Figure 1.

Before editing, the size of the *sigF* gene region in the genome of *B. pumilus* 3-19 was 1751 bp; after editing the genome, the length of the region was 1216 bp (Figure 1A). These results indicate that there was a deletion of the sporulation factor gene. The resulting mutant strain has the genotype *strR*, Δ*sigF*.

Bands ~1004 bp (*bact*) and ~806 bp (*bac*) corresponded to the lengths of the gene fragments that are contained in the plasmids pVYb11.21 (Figure 1B) and pDIb11.21 (Figure 1C), respectively. Before editing, the size of the *bact* gene region in the genome of *B. pumilus* 3-19 and *bac* gene was 1470 bp and 1651 bp, respectively. As a result of targeted inactivation of the *bac* and *bact* genes, mutant strains of *B. pumilus* 3-19 with inactive genes of antimicrobial peptides—bacilysin and bacteriocin, respectively—were obtained.

### 3.4. Antimicrobial Activity of B. pumilus 3-19 Strains

**Figure 2:**
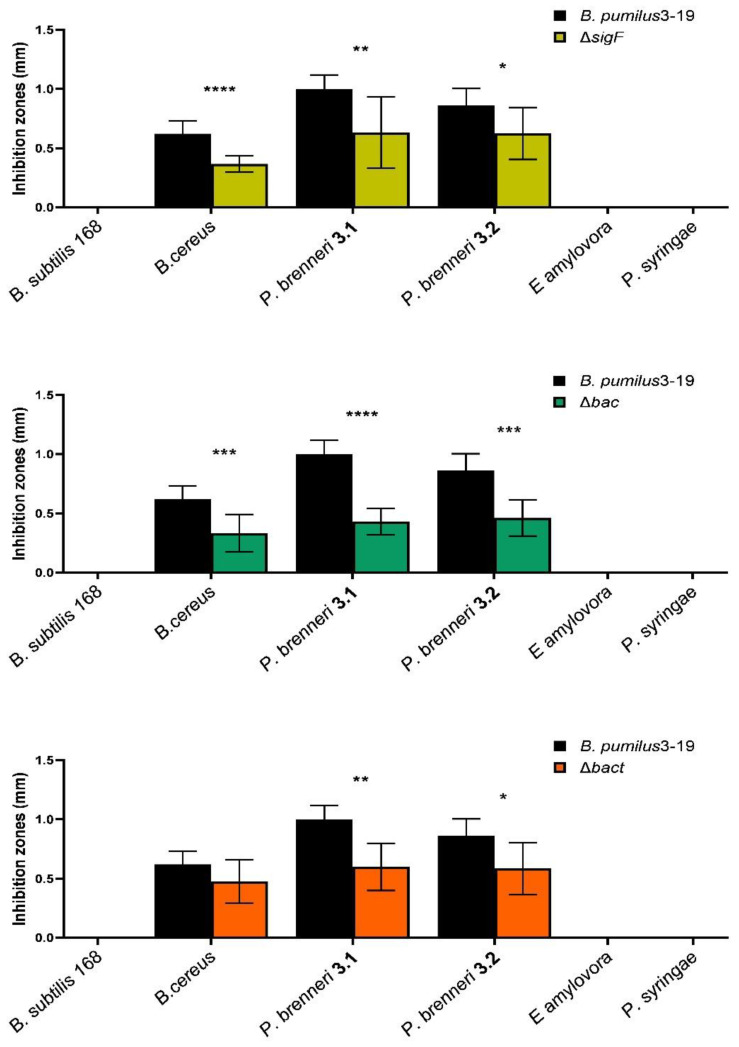
Antimicrobial activity of mutant strains derived from *B. pumilus* 3-19 in comparison with the native strain. * *p* ≤ 0.05, ** *p* ≤ 0.01, *** *p* ≤ 0.001, **** *p* ≤ 0.0001.

Determination of antimicrobial activity in mutant strains of *B. pumilus* 3-19 with inactivated genes was carried out using six strains of soil microorganisms isolated from various sources (Table 1). The study showed that the wild *B. pumilus* 3-19 strain exhibited antimicrobial activity against the test *B. cereus* and *P. brenneri* (3.1, 3.2) strains (Figure 2). The maximum antimicrobial effect was exerted on *P. brenneri* 3.1 (the zone of inhibition was 1.0 cm).

The spectrum of antimicrobial activity of the tested mutant strains changed. In particular, inactivation of the genes of antimicrobial peptides (*bac* and *bact*) resulted in a decrease in antimicrobial activity against *B. cereus* and *P. brenneri* (3.1, 3.2). At the same time, it was noted that the antagonistic activity of the Δ*bac* mutant strain (bacilysin gene removed) was lower compared to the Δ*bact* mutant strain (bacteriocin gene removed). Apparently, the contribution of the antimicrobial peptide bacillysin to the manifestation of the antagonistic activity of *B. pumilus* 3-19 is greater than that of bacteriocin.

Freitas-Silva et al. showed that *B. pumilus*, with the aid of bacilysin, is able to effectively suppress the potato pathogen *Phytophthora infestans* by 50% [30]. In addition, other representatives of the *Bacillus* genus—in particular, the bacterium *B. amyloliquefaciens*—are capable of producing this antimicrobial peptide which has shown antagonistic activity against opportunistic microorganisms *Malassezia furfur* and *Malassezia globosa* [31].

Bacycycline XIN-1 at a concentration of 25 μM completely inhibited the growth of *B. cereus* and significantly reduced the growth of *L. monocytogenes* and *S. aureus* in 7 days [32].

**Figure 3:**
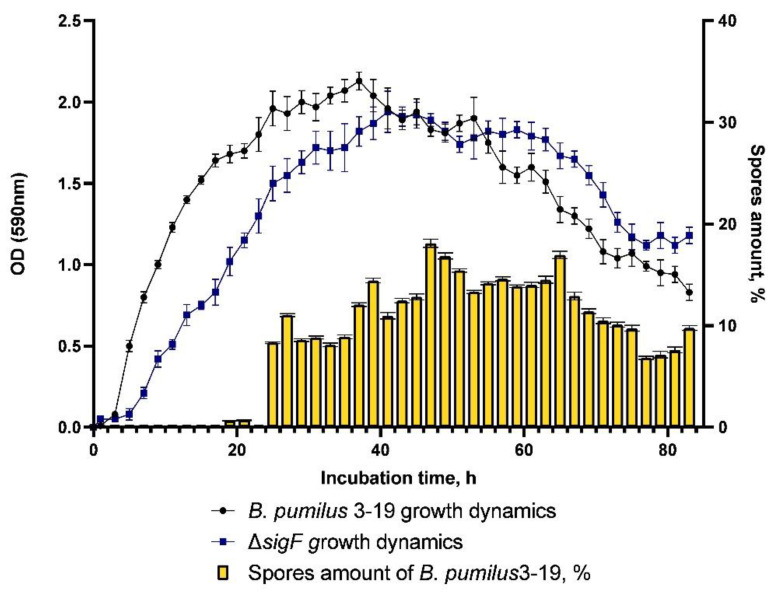
Dynamics of growth and dynamics of spore formation of *B. pumilus* 3-19 and mutant strain *B. pumilus* 3-19 (Δ*sig*F).

Wang et al. showed that deletion of the *spo*0A gene (the main regulator of sporulation entry) led to a sharp decrease in bacilysin synthesis [33]. It was also demonstrated that the *sig*F mutation has minimal effect on the transcriptome under vegetative conditions: 117 genes were expressed differently in the *sigF* mutant strain compared to the wild-type strain; in particular, the bacteriocin gene was among the genes with reduced expression. The level of its expression in the mutant decreased by 2.67 times compared to the wild strain [34]. These data suggest that the sporulation process is interrelated with the production of antimicrobial peptides. In our study, the sporulation factor gene *sigF* was inactivated. The mutant strain Δ*sigF* was found to be asporogenic. The dynamics of sporulation of the control strain *B. pumilus* 3-19 is characterized by the presence of spores from the 25th hour onwards; the maximum number of spores was 19%, at the 47th hour (Figure 3).

It was discovered that the antagonistic activity of the *sigF* mutant strain against *B. cereus* and *P. brenneri* (3.1, 3.2) decreased by 42, 37, and 27%, respectively (Figure 2). Judging by the fact that this strain exhibited antagonistic activity similar to the mutant strain with the deletion in the *bac* gene, it can be assumed that the deletion of the *sigF* gene led to a decrease in the synthesis of bacilysin.

In relation to other soil bacteria—*B. subtilis* 168 and two phytopathogens *E. amylovora* and *P. syringae*—antagonistic activity was not manifested in any of the studied strains of *B. pumilus* 3-19. However, growth retardation was observed in both phytopathogens under the action of the native *B. pumilus* 3-19 strain and the Δ*bact* mutant. These data indicate the contribution of *B. pumilus* 3-19 bacilysin to the manifestation of antagonistic activity against soil bacteria. Thus, we found that the antimicrobial activity of recombinant strains is lower compared to the activity of the native strain. 

### 3.5. Growth Dynamics and Proteolytic Activity of B. pumilus 3-19 Strains

**Figure 4:**
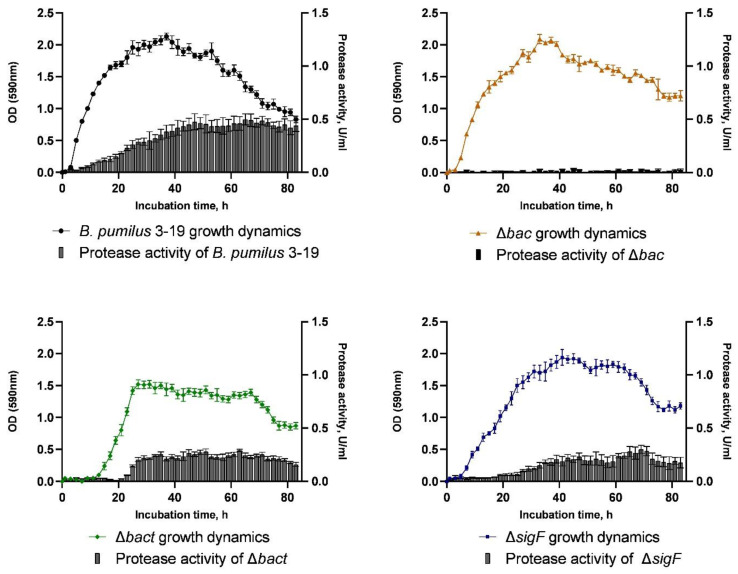
Dynamics of growth and proteolytic activity of native strain *B. pumilus* 3-19 and its mutant strains.

The growth dynamics of *B. pumilus* 3-19 and mutant strains was measured for 83 h (Figure 4). As a result, the wild strain *B. pumilus* 3-19 reached the maximum value of optical density equal to OD_590_ = 2.13 at the 37th hour of cultivation. At the same time, the optical density of the mutant strain *B. pumilus* 3-19 (Δ*bac*) was close to the growth dynamics of *B. pumilus* 3-19, and the maximum number of cells was reached at the 33^rd^ hour of culture growth (OD_590_ = 2.09).

In the mutant *B. pumilus* 3-19 (Δ*bact*) strain, the lag phase was prolonged and ended by the 15th hour of cultivation. The biomass of *B. pumilus* 3-19 (Δ*bact*) reached its maximum value at the 27th hour (OD_590_ = 1.52) and was significantly lower (by 29%) compared to the wild strain. Further, the culture growth enters the stationary phase.

The growth dynamics of *B. pumilus* 3-19 mutant in the *sigF* gene was characterized by an exponential phase lasting 31 h, after which, the stationary phase began. The maximum value of OD_590_ (1.85) was reached at the 41st hour of cultivation. The cell death phase began at the 65th hour. The growth dynamics of the mutant strain is characterized by a longer stationary phase (Figure 4).

The synthesis of extracellular proteinases by bacilli is related to peptide synthesis and sporulation [34]. In order to investigate the impact of target gene inactivation on the production of extracellular proteinases, hydrolysis of azocasein was used to measure proteolytic activity. Every 2 h for 83 h, the presence of enzymes in the culture medium was assessed (Figure 4). According to the studies, the wild strain *B. pumilus* 3-19 showed the highest level of proteolytic enzyme activity at the 65th hour of cultivation (0.498 U/mL). The extracellular enzyme activity in the *bac* and *bact* mutant strains was 0.027 U/mL and 0.291 U/mL, respectively, lower than in the wild type. We have shown that the secretion of proteinases in *B. pumilus* 3-19 decreases upon inactivation of the antimicrobial peptide genes. For the mutant strain Δ*sigF*, the maximum proteolytic activity occurred by 65 h and was 0.280 U/mL. This indicates a decrease in the proteolytic activity of the mutant strain by 53%.

By mutating *sigF*, unwanted sporulation is avoided for various experiments such as industrial fermentation, long-term experimental evolution, or null growth studies. There is evidence that disruption of *spo*IIAC (*sigF*) increases resistance to spore formation and increases secretion of β-cyclodextrin-glycosyltransferase into the extracellular environment in *B. subtilis* [34]. Zhou et al. showed that the greatest effect on increasing the synthesis of alkaline protease was found in the *B. licheniformis* mutant with the removal of *sigF* [35].

Thus, in our study, inactivation of target genes resulted in a decrease in proteolytic activity in mutant strains compared to the wild type strain.

## 4. Conclusions

Since the antimicrobial peptides produced by bacilli are known to be able to limit the growth or activity of pathogenic bacteria, these strains may be useful in the bacterial–plant relationship [36].

In this work, the *B. pumilus* 3-19 strain was studied for its antagonistic activity against soil bacteria including phytopathogens. In order to determine the role of a specific antimicrobial peptide synthesized by the *B. pumilus* 3-19 strain, the genes for antimicrobial peptides (bacilysin (*bac*) and bacteriocin (*bact*)) and the gene for the second stage of sporulation spoIIAC (*sigF*), encoding the sigma factor F, were inactivated in its genome. 

Based on the shuttle vector pJOE9282.1, plasmids pDIb11.21, pVYb11.21, and pGAs11.21 were constructed to carry components of the CRISPR/Cas9 system, spacer fragments (sgRNA), and target regions of target genes, bacilysin (*bac*-L and *bac*-R), bacteriocin (*bact*-L and *bact*-R), and sporulation factor (*sigF*-L and *sigF*-R), respectively. The resulting vectors were used for transformation of *B. pumilus* 3-19 cells. Targeted inactivation of the bacilysin (*bac*), bacteriocin (*bact*), and sporulation factor (*sig*F) genes in the *B. pumilus* 3-19 genome was carried out using editing by CRISPR/Cas9 technology.

It was shown that antimicrobial peptides, such as bacilysin and bacitracin, can act as a signal molecule and affect many cellular functions, indirectly affecting growth dynamics [11,37,38].

It was found that the inactivation of antimicrobial peptide genes in the *B. pumilus* 3-19 genome led to a change in the growth dynamics and a decrease in the proteolytic activity of the edited strains (18.4 times for Δ*bac* and 2 times for Δ*bact*), as well as a decrease in antimicrobial activity in relation to *B. cereus* and *P. brenneri* (3.1, 3.2). It was noted that, apparently, the contribution of the antimicrobial peptide bacilysin to the manifestation of the antagonistic activity of *B. pumilus* 3-19 is greater than that of bacteriocin.

An asporogenic mutant strain was obtained by inactivating the sigma factor gene *(sig*F). The proteolytic activity of the recombinant strain *B. pumilus* 3-19 (Δ*sigF*) was 53% lower than that of the native strain; however, it was preserved. This strain exhibited antagonistic activity similar to the mutant strain in the *bac* gene, indicating that the deletion of the *sig*F gene led to a decrease in the synthesis of bacilysin.

In relation to the phytopathogens *E. amylovora* and *P. syringae*, antagonistic activity was not manifested in any of the studied strains of *B. pumilus* 3-19. However, under the influence of the native strain *B. pumilus* 3-19 and the Δbact mutant, growth retardation was observed in both phytopathogens. This indicates a special role of *B. pumilus* 3-19 bacilysin in its antagonistic activity against soil bacteria.

In the future, we plan to elucidate the mechanism of the pleiotropic action of peptides of different natures as a signal molecule, describe the physiological and metabolic processes that these molecules act on, and conduct a comparative characterization of phenotypes and transcriptomic analysis of mutant strains in order to use targeted compositions with a more pronounced antimicrobial effect in practice.

## Figures and Tables

**Table 1:** Bacterial strains used in the work.

Strain	Genotype	Purpose of Use	Source
*Bacillus pumilus* 3-19 [17]; Genbank accession number NZ_CP054310	*strR*	Gene deletion, recipient strain	Laboratory collection (Department of Microbiology, “Agrobioengineering” research laboratory, KFU)
*Escherichia coli* DH5α; https://www.ncbi.nlm.nih.gov/nuccore/NZ_CP076470.1, (accessed on 20 March 2022)	–	Obtain recombinant plasmids	
*Pantoea brenneri* 3.1, 3.2 (all-Russian collection of industrial microorganisms B-12911) [18]	–	Antagonistic activity	
*B. subtilis* 168; https://www.ncbi.nlm.nih.gov/Taxonomy/Browser/wwwtax.cgi?mode=Info&id=224308&lvl=3&lin=f&keep=1&srchmode=1&unlock, (accessed on 17 March 2009)		Antagonistic activity	
*B. cereus*	–	Antagonistic activity	
*P. syringae;* https://www.ncbi.nlm.nih.gov/Taxonomy/Browser/wwwtax.cgi?mode=Info&lvl=3&lin=f&keep=1&srchmode=1&unlock&id=323, (accessed on 1 November 2022)	–	Antagonistic activity	Plant Infectious Diseases Laboratory, Kazan Scientific Center of Russian Academy of Sciences
*Erwinia amylovora*	–	Antagonistic activity	All-Russian research institute of phytopatology
*B. pumilus* AG11.21	*strR*, Δ*sigF*	A strain with an inactivated *sigF* sporulation factor gene	Received in the work
*B. pumilus* ID11.21	*strR*, Δ*bac*	A strain with an inactivated bacilysin gene	Received in the work
*B. pumilus* IV11.21	*strR*, Δ*bact*	A strain with an inactivated bacteriocin gene	Received in the work

**Table 2:** Primers for hybridization of spacer fragments.

No	Primer Name	Sequence 5′→3′
1	*bac*-sgRNA-For	ctcgATTTCAAGAACAAATGC
2	*bac*-sgRNA-Rev	aaacGCATTTGTTCTTGAAAT
3	*bact*-sgRNA-For	ctcgTCTCGCTACAGATGCTG
4	*bact*-sgRNA-Rev	aaacCAGCATCTGTAGCGAGA
5	*sigF*-sgRNA-For	ctcgTCGGCTATTTCCTGGACAGT
6	*sigF*-sgRNA-Rev	aaacACTGTCCAGGAAATAGCCGA

**Table 3:** Oligonucleotides used in the work.

No	Primer Name	Sequence 5′→3′ *
1	*bac*L-For	aaGGCCaacgaGGCCTTTTCTTAGTGCTGGGCGGC
2	*bac*L-Rev	aaGGCCatgttGGCCGCACATACCTAGATGTGCAGTGA
3	*bac*R-For	aaGGCCaacatGGCCCCCTCCTCACACGATCATAGAAC
4	*bac*R-Rev	aaGGCCttattGGCCCCGCTGATGCAAATGCCGATT
5	*bact*L-For	aaGGCCaacgaGGCCCATCAAGACTTGCCTACTCCCT
6	*bact*L-Rev	aaGGCCatgttGGCCTGAGAAAAGAATCGTTTTTGCTAGG
7	*bact*R-For	aaGGCCaacatGGCCTGACTCAATAGAAGGAGACTTTGTT
8	*bact*R-Rev	aaGGCCttattGGCCATGTGTTTGTGCAATGGAATATTGA
9	*sigF*L-For	aaGGCCaacgaGGCCAGCCATACAAACACAAATAAAACGG
10	*sigF*L-Rev	aaGGCCatgttGGCCACAAGTACAAGTCTCGCGGC
11	*sigF*R-For	aaGGCCaacatGGCCAAAAGCGCTGAACAACGGAC
12	*sigF*R-Rev	aaGGCCttattGGCCTATCTCGCCAGCAGTGAACC
13	Cas9 For	CGCGTGGCAATAGTCGTTTT
14	Cas9 Rev	ATGCCGCCCCATTACTTTGA
15	T7promoter For	TAATACGACTCACTATAGGG

Note: * restriction sites are indicated in lowercase letters.

## Data Availability

The data presented in this study is contained within this article.

## Supplementary Materials

The following supporting information can be downloaded at: https://www.mdpi.com/article/10.3390/microorganisms11061508/s1, Figure S1: Schematic representation of pJOE9282.1 plasmid: R, L—sites of insertion of bacilysin, bacteriocin, and sigma-F sporulation factor genes fragments at the SfiI site; Figure S2. Electrophoresis of amplification products of gene fragments: M—DNA marker (10 Kb); 1—*bac*-L (404 bp); 2—*bac*-R (402 bp); 3—*bact*-L (501 bp); 4—*bact*-R (503 bp); 5—*sigF*-L (500 bp); 6—*sigF*-R (716 bp); Figure S3.—Colonies of transformed *B. pumilus* 3-19 cells. C—negative control; 1—colonies of *B. pumilus* 3-19 transformants with pDIb11.21 vector; 2—colonies of *B. pumilus* 3-19 transformants with pVYb11.21 vector; 3—colonies of *B. pumilus* 3-19 transformants with pGAs11.21 vector.
