# Supplementary material for: Characterization of Bacillus pumilus Strains with Targeted Gene Editing for Antimicrobial Peptides and Sporulation Factor
Source: Microorganisms. 2023 Jun 6;11(6):1508. doi: 10.3390/microorganisms11061508 (PMC10303315; doi:10.3390/microorganisms11061508)
Supplement: Supplementary file 1 [file microorganisms-11-01508-s001.zip › microorganisms-2400539-supplementary.pdf]

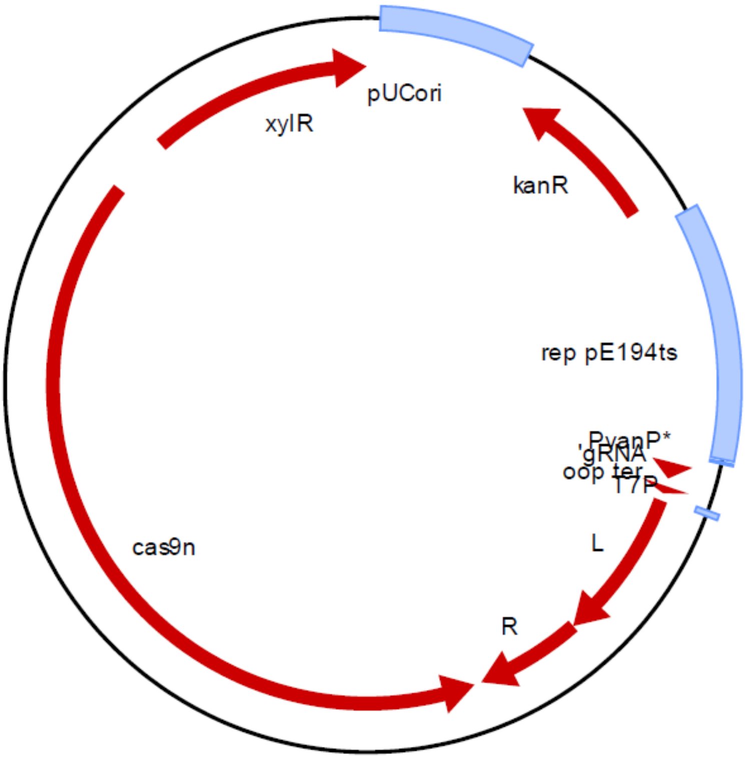

Figure S1. Schematic representation of pJOE9282.1 plasmid: R, L – sites of insertion of bacilysin, bacteriocin, and sigma-F sporulation factor genes fragments at the SfiI site

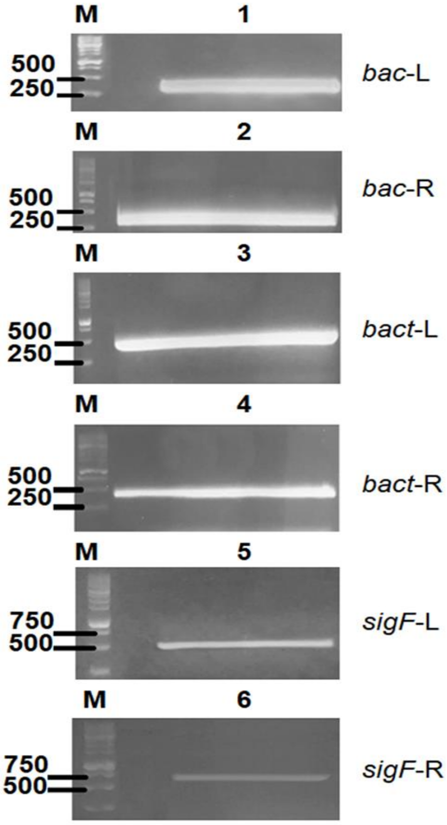

Figure S2. Electrophoresis of amplification products of gene fragments: M - DNA marker (10 Kb); 1 – *bac*-L (404 b.p.); 2 – *bac*-R (402 bp); 3 – *bact*-L (501 b.p.); 4 – *bact*-R (503 b.p.); 5 – *sigF*-L (500 b.p.); 6 – *sigF*-R (716 b.p.).

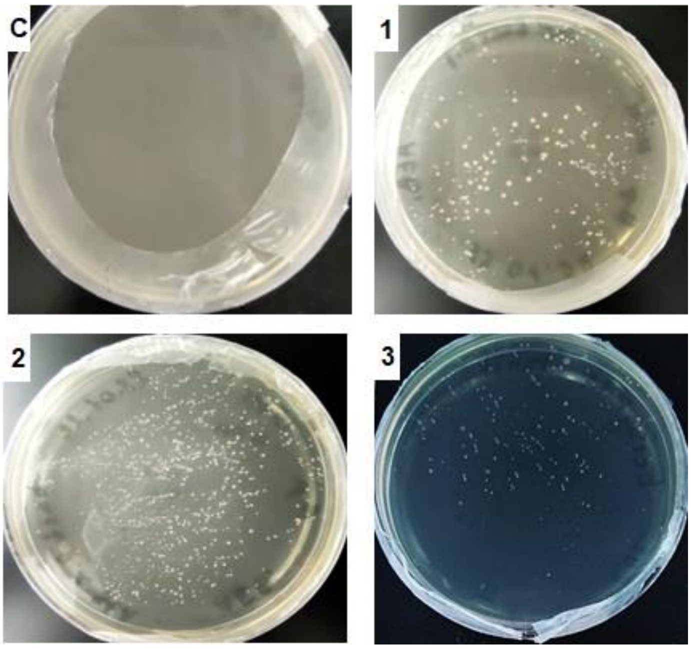

Figure S3. - Colonies of transformed *B. pumilus* 3-19 cells. C - negative control; 1 – colonies of *B. pumilus* 3-19 transformants with pDIb11.21 vector; 2 – colonies of *B. pumilus* 3-19 transformants with pVYb11.21 vector; 3 – colonies of *B. pumilus* 3-19 transformants with pGAs11.21 vector.
